# Sexual Functioning in Individuals With Attention-Deficit Hyperactivity Disorder: A Narrative Review

**DOI:** 10.7759/cureus.97194

**Published:** 2025-11-18

**Authors:** Agata Puszcz, Martyna Platnikow, Zuzanna Antos, Marta Czech, Anna Kipka

**Affiliations:** 1 Department of Clinical Psychology, Poznan University of Medical Sciences, Poznań, POL

**Keywords:** attention-deficit disorder with hyperactivity, hypersexuality, paraphilic disorders, sensory processing disorder, sexual dysfunctions

## Abstract

This narrative review aimed to provide a synthetic overview of current knowledge on the relationship between attention-deficit hyperactivity disorder (ADHD) and sexual functioning in adults, with a particular emphasis on neurobiological mechanisms, comorbid disorders, and the impact of pharmacotherapy.

Six independent researchers conducted a comprehensive literature review using the PubMed, Scopus, and Google Scholar databases, encompassing publications available as of May 2024. Original research papers, reviews, and meta-analyses in English and Polish concerning adult populations with ADHD were included. Pediatric studies and papers published before 2014 were excluded.

Data indicate that a substantial proportion of men and women with ADHD experience sexual dysfunctions. Their nature is heterogeneous and dependent on the individual manifestation of the disorder, encompassing both hypersexuality and hyposexuality. Predisposing factors include sensory integration disorders, attention deficits, hyperfocus, and a need for frequent changes. An important role is also played by rejection-sensitive dysphoria (RSD), which is associated with a high prevalence of risky sexual behaviors and difficulties in maintaining relationships. Epidemiological studies suggest an increased prevalence of compulsive sexual behavior disorder (CSBD) and problematic pornography use in individuals with ADHD, particularly men. Some authors emphasize the importance of neurobiological mechanisms such as “dopaminergic craving” or novelty-seeking, while others indicate that hypersexual behaviors may be treated as a form of self-regulation. Concerning pharmacotherapy, psychostimulant drugs (e.g., methylphenidate and lisdexamfetamine) and nonstimulants (e.g., atomoxetine and clonidine) demonstrate both beneficial and adverse impacts on sexual functioning, although empirical data are limited. ADHD shows a significant correlation with the occurrence of a wide spectrum of sexual disorders, which requires consideration in diagnostics and treatment planning. The high prevalence of hypersexuality, CSBD, and RSD highlights the need for an interdisciplinary approach combining pharmacological interventions with psychotherapy focused on emotional regulation, improvement of interpersonal relationships, and reduction of risky behaviors. Further research should focus on identifying neurobiological mechanisms, the role of psychosocial factors, and evaluating the impact of pharmacotherapy on the sexuality of patients with ADHD.

## Introduction and background

According to the Diagnostic and Statistical Manual of Mental Disorders, Fifth Edition (DSM-5) criteria, attention-deficit hyperactivity disorder (ADHD) is characterized by a persistent pattern of attention deficits and/or excessive activity-impulsivity that exceeds the normal variability expected for age and level of intellectual functioning and has been present for at least six months. For children up to 16 years of age, a minimum of six symptoms in a given domain is required; for adolescents aged 17 years and older and adults, at least five symptoms are required. These symptoms manifest in various situations and areas of life (e.g., at work and during social interactions), indicating pervasiveness. There must be clear evidence that the symptoms interfere with, or reduce the quality of, social, academic, or occupational functioning, and the presentation should not be better attributed to other mental, behavioral, or neurodevelopmental disorders, nor can the effects of substances or medications cause them. Currently, three subtypes are distinguished: ADHD-I (Inattentive Subtype), ADHD-H (Hyperactive-Impulsive Subtype), and ADHD-C (Combined Subtype) [1]. Research on the comorbidity of ADHD and sexual disorders has begun relatively recently. A sexual disorder entails a clinically significant disruption of sexual responsiveness or the experience of sexual pleasure (DSM-5) [1].

Our research team proceeded from the premise that the sexual domain may be affected by illness to the same extent as the occupational or emotional domains; therefore, to provide a comprehensive characterization of sexuality, we must look beyond simple comorbidity with sexual dysfunctions. We sought to prepare an integrated analysis that highlights all challenges in sexual life that patients with ADHD may encounter. In this characterization, we included not only pathology but also differences at the levels of sensory stimulus processing and the formation of stable romantic and sexual relationships.

The sexuality of ADHD is a topic that gained importance at the beginning of the 21st century, when awareness increased regarding ADHD as a disorder not only of childhood but also persisting into adulthood. In 2014, with the introduction of DSM-5, the official diagnostic criteria for ADHD were revised, and the disorder began to be coded as a neurodevelopmental disorder whose course may evolve across the lifespan. The criteria emphasized the disorder’s impact across multiple domains of adult functioning, including occupational and interpersonal spheres. Our objective was to synthesize the considerations of multiple research teams that have attempted to delineate the effects of this disorder on sexual functioning.

This article is a narrative review of the literature concerning the neurobiological aspects of ADHD and their relationship with sexual functioning. The paper aimed to provide a synthetic overview of existing empirical and theoretical findings based on current scientific knowledge, map ADHD-related alterations across sexual functioning domains, synthesize putative mechanistic pathways and the effects of pharmacotherapy, translate evidence into a conceptual framework and practical screening/counseling recommendations, and identify the main research areas and potential directions for further analysis. Despite growing interest, evidence remains fragmented by small samples, heterogeneous measures of sexual outcomes, and limited sex-/gender-stratified analyses. A consolidated synthesis that links mechanisms, treatments, and clinically actionable recommendations is lacking.

The etiology of ADHD involves a complex interaction of genetic and environmental factors. According to literature data, the heritability of ADHD may reach up to 80% [2]. The disorder is associated with hypoactivity of the frontal cortex and subcortical structures, resulting in reduced synthesis of dopamine and norepinephrine [3].

In ADHD, dysfunction of the dopaminergic system is observed, particularly within the mesocortical, mesolimbic, and nigrostriatal pathways [4]. The decreased dopamine level is explained by defects in the genes encoding dopamine receptors D4 and D5 [5]. Disturbances within the mesocortical pathway are associated with deficits in executive functions, including impaired concentration, planning, organization of actions, and cognitive control. Hypoactivity of the mesolimbic pathway is considered one of the leading causes of reward system dysfunction observed in individuals with ADHD, manifested by reduced motivation and an increased need for immediate gratification. In turn, abnormalities in the functioning of the nigrostriatal pathway, which is involved in both the control of voluntary movements and the regulation of cognitive processes, may account for the hyperactivity and impulsivity component characteristic of the clinical phenotype of ADHD [6]. Gadow et al. demonstrated that certain variants of genes associated with the dopaminergic system may modulate the severity of ADHD symptoms [7]. In a separate study, it was found that disturbances in dopaminergic transmission within the right caudate nucleus correlate with the severity of inattention symptoms and deficits in response inhibition in individuals diagnosed with ADHD. Based on this, the authors postulate that pharmacological interventions aimed at increasing tonic dopamine levels may represent an effective therapeutic strategy for treating this disorder [5].

Among the environmental factors contributing to the development of ADHD are, among others, fetal exposure to toxic substances such as nicotine and alcohol, perinatal stress, fetal hypoxia during delivery, intrauterine growth restriction, and prematurity [8]. The literature also indicates a significant influence of psychosocial factors such as low parental education level, environmental deprivation, experiences of violence, and peer victimization. It has been shown that rare genetic disorders such as neurofibromatosis type 1, Angelman syndrome, Prader-Willi syndrome, fragile X syndrome, tuberous sclerosis, and DiGeorge syndrome frequently co-occur with ADHD [9].

Saad et al. conducted a systematic review of neuroimaging studies comparing the ADHD-I and ADHD-C subtypes. The analysis included 19 studies that used structural magnetic resonance imaging (sMRI), functional magnetic resonance imaging (fMRI), and diffusion tensor imaging (DTI) to identify morphological and functional differences between these subtypes. The authors emphasize that existing sMRI studies do not provide consistent or unequivocal results regarding gray and white matter volumes in patients with ADHD-I and ADHD-C, and it is currently not possible to delineate stable morphometric differentiating markers. In contrast, findings from fMRI and DTI studies show more consistent differences at the level of functional network organization and microstructural integrity. Individuals with ADHD-C more frequently exhibit abnormalities within the frontostriatal thalamic network, which is involved in behavioral inhibition control, motor function, and reward processing. Conversely, individuals with ADHD-I demonstrate abnormalities in the cingulo-fronto-parietal and visual networks, which may be associated with predominant difficulties in maintaining attention and processing sensory information [10].

Stokholm et al. proposed a new direction in ADHD pathogenesis research, suggesting a possible link between prenatal exposure to synthetic oxytocin, administered to induce labor, and the risk of developing ADHD later in life in the fetus. The analysis revealed a statistically significant but small increase in ADHD risk (adjusted hazard ratio ~1.03). However, the authors emphasize that the observed association does not allow for causal inference, mainly due to the presence of numerous potential confounding variables. These findings simultaneously highlight the need for further studies employing more rigorous sample selection criteria, for example, limited to primiparous women [11].

## Review

Methodology

Five independent authors conducted a search of the medical databases PubMed, Scopus, and Google Scholar, using keywords and Medical Subject Headings terms: “sexual behavior” or “sexuality”, occurring in the article together with the headings “Attention Deficit Disorder with Hyperactivity” (178 results), “ADHD” (202 results), and “Neurodevelopmental Disorders” (640 results), in both English and Polish. The inclusion criteria comprised original articles, reviews, and meta-analyses published in English or Polish that concerned adults with a diagnosis of ADHD. Exclusion criteria comprised pediatric case reports, preprints (not yet peer-reviewed), articles published before 2014 (given the date of introduction of DSM-5 criteria on which the present review is based), and studies concerning pediatric populations, owing to the not yet fully established sexual preferences in this group of patients. The literature review was conducted from January to May 2024. The collected records were then assessed for compliance with the predefined inclusion and exclusion criteria based on abstract screening. Of the 1,020 articles initially retained for further assessment, 115 were excluded as duplicates, and 86 for failing to meet the above criteria. The remaining articles underwent abstract analysis, of which 104 publications were qualified for further, detailed evaluation. All included articles were assessed with respect to design, methods, type of intervention, therapeutic objective, and conclusions derived from the studies. Ultimately, 36 papers were included in the review. The aim of this study is also to indicate potential directions for optimizing therapeutic interventions and improving the quality of life of patients with ADHD.

The most common sexual dysfunctions in patients with ADHD

Sexual dysfunctions are observed in 39% of men and 43% of women diagnosed with ADHD [12]. The underlying mechanisms of these disorders are heterogeneous and depend on the individual clinical presentation of the disease. ADHD symptoms predisposing to the development of sexual dysfunctions include sensory integration disorders, attention deficits, the phenomenon of hyperfocus, and frequent changes in preferences and interests.

In patients with hypersensitivity to sensory stimulation, sensory stimuli may be perceived as excessively intense, uncomfortable, or painful. Such experiences can lead to the avoidance of initiating sexual contact, resulting in reduced sexual satisfaction and, in extreme cases, the development of sexual aversion. Sensory integration disorders in individuals with ADHD may manifest as both hyposensitivity and hypersensitivity to tactile, auditory, and visual stimuli. The severity of hypersensitivity ranges from mild discomfort to marked aversion and intense disgust toward certain smells, textures, or sounds. In some cases, even ordinary touch is perceived as a painful experience [13].

Attention deficits are also an important pathogenic factor. An excessive number of stimuli during sexual activity may result in difficulties maintaining an erection and sexual arousal. Conversely, rapid shifting of attention to other stimuli leads to instability in the level of sexual desire, comparable to mood instability observed in patients with ADHD. Consequently, both erectile and orgasmic disorders, as well as opposite phenomena such as premature ejaculation or hypersexuality, may develop. Attention deficits and the excessive intensity of perceived stimuli may also lead to reduced emotional presence during intercourse, posing a significant challenge to maintaining healthy partner relationships [13].

Periods of excessive concentration (hyperfocus) in individuals with ADHD may lead to engagement in activities that provide immediate gratification [12]. Current views on ADHD support the hypothesis that attention deficits may be context-dependent. This means that individuals with ADHD exhibit deficits in concentration and attention toward stimuli that are insufficiently attractive to them; at the same time, they are capable of long-term hyperfocus with reduced cognitive effort when the stimuli are highly interesting and rewarding [14]. In the context of intimate relationships, this phenomenon is particularly noticeable in the early stages of a partnership: a person diagnosed with ADHD may become excessively involved with their partner, manifested by intense attention, frequent gifts, abundant compliments, and heightened sexual interest [15]. Some partners of individuals with ADHD may feel overwhelmed by such behaviors, which highlights the importance of proper communication in maintaining balance within the relationship [15].

Results of studies conducted in 2022 indicate that there is no clear correlation between ADHD and paraphilias. Among 160 individuals diagnosed with ADHD, 47% were found to have sexual preference disorders, suggesting a predisposition rather than a direct causal relationship [16]. Conversely, other meta-analyses have shown that the prevalence of ADHD in the population of individuals with paraphilias is higher than in the general population [17]. Therefore, routine assessment of patients with paraphilias for ADHD symptoms is recommended, as this may help determine the potential effectiveness of ADHD treatment in reducing co-occurring sexual dysfunctions.

In the case of women with ADHD, the authors distinguish two sexual functioning styles: the “physical” and the “emotional.” The physical style may be associated with excessive sexual demands, whereas the emotional style focuses primarily on the need for emotional security, which may consequently limit the ability to experience sexual desire. The literature also emphasizes that individuals with ADHD, due to deficits in planning and sustaining attention, often devote insufficient time to nurturing intimacy in their relationships, which may lead to decreased sexual satisfaction [12].

Hypersexuality and compulsive sexual behavior disorder

The term “hypersexuality” was introduced into the scientific literature in 2010 by Kafka and Hennen and represented one of the academic proposals emphasizing the need to develop official diagnostic criteria for the condition currently classified as compulsive sexual behavior disorder (CSBD) [18]. As demonstrated by Gola et al., hypersexuality should be understood as a psychopathological syndrome that co-occurs with many psychiatric disorders but rarely appears as a separate nosological entity. In recent years, there has been a significant evolution in the understanding of the mechanisms underlying this phenomenon, as well as in diagnostic criteria, therapeutic approaches, and its nosological classification. Historically, the concept of hypersexuality appeared as “nymphomania” in reference to women and “satyriasis” in reference to men, while in the 10th edition of the International Classification of Diseases (ICD-10), it was categorized under code F52.7 as “excessive sexual drive.” With the publication of ICD-11, the approach to classifying this condition was modified, and it is now included in the category of impulse control disorders within the CSBD spectrum [19].

The diagnostic criteria for CSBD include spending a large amount of time on sexual fantasies or behaviors; recurrent sexual behaviors or fantasies in response to stressful life events and dysphoric emotional states (anxiety, sadness, boredom); habitual neglect of other important goals, activities, and obligations not directly related to sex; unsuccessful attempts to control or significantly reduce sexual behaviors or fantasies; repeated instrumental sexual behaviors despite negative consequences and a progressively lower level of resulting satisfaction; feelings of guilt; and emotional distress. Since CSBD has only recently been recognized as a distinct disorder, we use the terms “hypersexuality” or “compulsive sexual behaviors” when referring to studies published before 2022, and “CSBD” for more recent research [20].

The increased prevalence of hypersexuality in the population of patients with ADHD finds a potential explanation within the framework of the hypersensitivity-to-stimulation hypothesis. This mechanism is associated with dysfunction of the reticular formation and a dopaminergic deficit, referred to as “dopamine hunger,” which is characteristic of this disorder [21]. In individuals with ADHD, strong stimuli that activate the reward system, such as erotic content, pornography, or exposure to nudity, as well as emotionally negative stimuli affecting the amygdala (e.g., stress, tension, anxiety, or dysphoria), are processed with excessive intensity due to the ineffective inhibitory mechanism of the reticular formation. Consequently, this group experiences a more rapid activation of pathological functional couplings involving the prefrontal cortex, nucleus accumbens, amygdala, and orbitofrontal cortex [22].

Patients with ADHD who exhibit pronounced hypersexual behaviors demonstrate a reduced ability to inhibit responses to erotic stimuli [23]. This results in easier and shallower sexual arousal and a weakened mechanism for extinguishing sexual drive after orgasm. At the same time, disturbances in dopaminergic transmission within the reward system impair the ability to delay gratification, leading to intensified sexual fantasies and compulsive actions aimed at short-term increases in dopamine levels and reduction of emotional tension. Over time, this may lead to loss of control over sexual behavior, excessive engagement in masturbation, consumption of pornographic content, and fantasizing, which in turn results in deterioration of interpersonal relationships, psychological distress, and an intensified sense of guilt [5,24].

The increased susceptibility of individuals with ADHD to the development of CSBD may also be explained by the so-called “novelty-seeking” mechanism. Stimuli associated with intense activation of the reward system, particularly those characterized by novelty or a strong rewarding value, are permanently encoded in limbic structures such as the nucleus accumbens and hippocampus and are later spontaneously recalled during states of dopaminergic deficit typical of ADHD [25].

Some researchers suggest an alternative explanation for the frequent co-occurrence of CSBD and ADHD: there is evidence that excessive, instrumental sexual behaviors may serve as a form of “self-medication” through the natural elevation of dopamine levels in synaptic clefts [26,27]. In this view, frequent sexual activity functions as a substitute for psychostimulant-based pharmacotherapy. However, this mechanism is regarded as a dysfunctional method of emotion regulation and stress coping, which only temporarily alleviates the symptoms of the disorder [26].

Other theories propose the opposite relationship: hypersexuality is not a form of self-medication but a possible side effect of chronic use of psychostimulant drugs such as methylphenidate. However, this theory does not account for cases of hypersexuality in patients with ADHD who have not undergone pharmacological treatment [21].

One of the most frequently observed forms of compulsive sexual behavior is problematic pornography use (PPU). Empirical data suggest that men with ADHD are more predisposed to excessive pornography use than women [24]. Pornography constitutes an easily accessible and rapidly acting stimulus that may temporarily compensate for “dopamine hunger.” From a clinical perspective, it is important to note that some symptoms of PPU may be secondary to prefrontal cortex hyperactivity. A high intensity of PPU among men may lead to increased difficulties in establishing and maintaining close relationships, as well as to a decrease in overall quality of life [28].

The research team of Bőthe et al. conducted multidimensional structural equation modeling on a sample of adult patients with ADHD (N = 14,043). They examined the relationships between the presence of ADHD, hypersexuality, and PPU. The study demonstrated that ADHD symptoms had positive and moderate associations with hypersexuality in both men and women (β = 0.50, p < 0.01; β = 0.43, p < 0.01, respectively). Among men, ADHD symptoms showed a positive and moderate relationship with PPU (β = 0.45, p < 0.01), whereas among women, the relationship between ADHD symptoms and PPU was positive but weak (β = 0.26, p < 0.01). This indicates that ADHD predisposes individuals to hypersexuality and, in men, also to pornography addiction (which was not observed among women) [21].

The association between ADHD and hypersexuality has also been demonstrated by other research teams, who estimated the prevalence of this comorbidity to be between 17% and 29.4% [17]. The comorbidity rate with PPU in the male population was estimated at 3%, representing a statistically significant but weak association. The researchers emphasize that many respondents may be compulsive yet undisclosed pornography users who do not seek help but meet the criteria for PPU [21].

Rejection-sensitive dysphoria

One of the common symptoms observed in individuals with ADHD in the context of romantic relationships is rejection-sensitive dysphoria (RSD). This term is defined as an intense, subjective fear of rejection, criticism, or harassment, regardless of whether such situations occur objectively. The result is severe emotional pain associated with a sense of failure to meet one’s own or others’ expectations [29]. Individuals affected by RSD constantly anticipate the possibility of rejection, excessively focus on its signs, and react to them with heightened emotional intensity [30]. These reactions serve as defense mechanisms and may manifest as aggression, anxiety, or social withdrawal [31].

Epidemiological data indicate that RSD symptoms may affect up to 99% of individuals with ADHD, with approximately one-third of patients considering them the most burdensome aspect of the disorder [29]. The research team of Bondü and Esser notes that RSD does not occur in all individuals with ADHD and may be overdiagnosed [31]. Importantly, each subsequent instance of rejection exacerbates RSD symptoms. A characteristic feature is anticipatory anxiety related to rejection, along with the tendency to overlook signs of acceptance from a partner in favor of excessive focus on perceived indicators of rejection [30]. Other symptoms of RSD include low self-esteem, anxiety in social situations, a tendency to feel embarrassed, difficulties in maintaining relationships, setting excessively high standards for oneself, and self-destructive thoughts [29].

Individuals with RSD often exhibit behaviors characterized by insecurity, restraint, and introversion in interpersonal relationships. This suggests a tendency to avoid rejection through distancing and limited engagement in intimate relationships. At the same time, these individuals experience a strong need for closeness, which leads to a paradox: attempts to protect themselves from rejection may be interpreted by others as a lack of interest and, in turn, trigger feelings of rejection themselves [30].

Research indicates that fear of intimacy among individuals with ADHD is heightened and correlates with the number of ADHD symptoms, feelings of inadequacy, previous experiences of rejection, and risky sexual behaviors, particularly in the ADHD-I subtype [26]. Difficulties in forming attachments and a lack of adequate support may contribute to the occurrence of hypersexual behaviors.

Van Eck et al. emphasize that rejection sensitivity may be considered a significant component of distress intolerance in individuals with ADHD. Consequently, such individuals are more likely to engage in casual sexual encounters as a means of avoiding the anticipated distress associated with rejection. Research findings indicate that the coexistence of ADHD and distress intolerance significantly increases the risk of engaging in risky sexual behaviors [32].

In the therapeutic context, Bondü and Esser developed recommendations for psychotherapists working with patients with ADHD. These include helping patients understand that their partners’ decisions and reactions may result from causes other than rejection or abandonment, as well as highlighting that patients’ own behaviors may contribute to the emergence of undesirable responses from others. Such strategies may help reduce impulsive and negative reactions to perceived rejection [31]. Furthermore, standard pharmacological treatment for ADHD has shown potential effectiveness in reducing RSD symptoms [29].

Sexual dysfunctions related to pharmacotherapy

Current pharmacotherapy for attention-deficit hyperactivity disorder focuses on reducing the most common core symptoms. Three main groups of drugs are distinguished for this purpose: psychostimulants, nonstimulants, and α2-adrenergic agonists. In clinical practice, the most commonly used substances are methylphenidate, a psychostimulant, and atomoxetine, a nonstimulant [33].

Both methylphenidate and atomoxetine affect sexual arousal through modulation of the mesolimbic-mesocortical system, resulting in enhanced dopaminergic transmission. Activation of dopamine receptors plays an important role in inducing spontaneous penile erections in men. These effects may be subjectively evaluated by patients as either beneficial or adverse [34].

Most available scientific reports and case descriptions concerning the adverse effects of methylphenidate refer to men, particularly to children and adolescent boys. Between 1997 and 2012, 15 cases of priapism were reported, occurring even with low doses of the drug (10 mg). The condition usually appeared independently of sexual arousal and caused pain ranging from significant to mild. This symptom typically occurred in the morning, shortly after waking, and could last for up to one hour [35].

In 2014, the results of a study were published analyzing the effects of methylphenidate and 3,4-methylenedioxymethamphetamine on the perception of sexual stimuli and functioning within intimate relationships. According to the obtained data, administration of methylphenidate was associated with an increased level of subjectively experienced sexual arousal among adult participants. At the same time, no significant effect of the drug on the quality of romantic relationships was observed. It is worth noting that relatively high doses of methylphenidate (40 mg) administered intravenously were used in this study [34].

In contrast to methylphenidate, atomoxetine belongs to the group of nonstimulant agents. The most frequently observed adverse effects related to sexual functioning include decreased libido and erectile dysfunction. The literature also describes a case of a 24-year-old patient who experienced spontaneous ejaculations during atomoxetine therapy. These occurred two to three times per day, accompanied by the sensation of orgasm, yet without any sexual stimuli [36].

Currently, pharmacological strategies involving off-label drug use are increasingly employed in the treatment of ADHD. One of the main representatives of this group is bupropion, a drug widely used in psychiatry, including in the treatment of depression and nicotine dependence. Reports on the use of bupropion in patients with ADHD first appeared in the 1980s, whereas its potential impact on sexual functioning became the subject of analysis in the following decade. At present, the literature does not contain unambiguous case descriptions regarding the influence of bupropion on sexual behaviors in patients treated with this medication. Nevertheless, an increased incidence of sexual dysfunctions has been observed in patients taking bupropion in combination with certain antidepressants, such as fluoxetine or venlafaxine [37].

The first studies on the effects of lisdexamfetamine dimesylate began to appear less than 20 years ago. The articles published so far have focused primarily on comparing this drug with commonly used preparations for the treatment of ADHD. Currently, few sources describe the adverse effects of this medication. However, reports have appeared in the literature suggesting potential benefits in the treatment of premature ejaculation in men [38].

Clonidine, belonging to the group of nonstimulant medications, was approved for ADHD therapy in 2010 [39]. At present, there are no reports in the literature describing sexual dysfunctions as adverse effects of clonidine.

## Conclusions

The reviewed literature highlights the complex and multifactorial relationship between ADHD and sexual functioning. Neurobiological mechanisms, particularly dopaminergic dysregulation and altered activity within the reward system, play a crucial role in the occurrence of sexual dysfunctions, including hypersexuality and compulsive sexual behaviors. Context-dependent attention deficits (including hyperfocus on the new relationship), sensory hypersensitivity, impulsivity, and RSD may further contribute to difficulties in maintaining romantic and sexual relationships.

An additional factor influencing the sexual functioning of patients with ADHD is the role of pharmacotherapy. It has been shown that first-line stimulant medications may cause disturbances in libido and erectile function. Due to dynamic changes in therapeutic strategies, including the use of alpha-blockers and antidepressants, as well as the introduction of new drugs to the market (such as lisdexamfetamine dimesylate), the number of side effects affecting sexuality is increasing. Considering the adverse impact of certain medications on sexual functioning, along with an individualized approach to the patient’s pharmacotherapy, may help maintain better compliance and enhance patient satisfaction with treatment.

A limitation of our review is that we did not have the scope to include an in-depth analysis of individual case reports, which could have provided additional, context-specific insights. We recognize this as an important constraint and intend to address it in a subsequent study. In future work, we plan to incorporate targeted case series to better capture heterogeneity across diverse backgrounds. Future research should focus on identifying specific predictors of sexual dysfunctions in this population of patients, developing standardized diagnostic criteria, and establishing targeted therapeutic strategies that address both ADHD symptomatology and sexual well-being. Increased clinical awareness of these interactions is essential to ensure comprehensive, individualized treatment and to improve the overall quality of life for patients affected by ADHD.
